# Antimicrobial treatment improves tryptophan metabolism and mood of patients with small intestinal bacterial overgrowth

**DOI:** 10.1186/s12986-022-00700-5

**Published:** 2022-09-27

**Authors:** Cezary Chojnacki, Tomasz Popławski, Paulina Konrad, Michał Fila, Janusz Błasiak, Jan Chojnacki

**Affiliations:** 1 Department of Clinical Nutrition and Gastroenterological Diagnostics, Medical University of Lodz, Haller square 1, 90-647 Lodz, Poland; 2 Department of Pharmaceutical Microbiology and Biochemistry Medical, University of Lodz, 90-136 Lodz, Poland; 3 Department of Developmental Neurology and Epileptology, Polish Mother Memorial Hospital Research Institute, 93-338 Lodz, Poland; 4 Department of Molecular Genetics, Faculty of Biology and Environmental Protection, University of Lodz, 90-236 Lodz, Poland

**Keywords:** Small intestinal bacterial overgrowth, Tryptophan metabolism, Anxiety, Depression, Rifaximin

## Abstract

**Background:**

Optimal composition of intestinal bacteria is an essential condition for good health. Excessive growth of these bacteria can cause various ailments. The aim of this study was to assess the mental state and gastrointestinal complaints of patients with small intestinal bacterial overgrowth (SIBO) in relation to tryptophan metabolism and rifaximin treatment.

**Methods:**

120 subjects, aged 23–61 years, were enrolled in the study, and divided into 3 groups, 40 individuals each: healthy subjects (Controls), patients with SIBO and chronic diarrhea (SIBO-D), and with chronic constipation (SIBO-C). The lactulose hydrogen breath test (LHBT) was performed to diagnose SIBO. The mental state of patients was assessed using the Hamilton Anxiety Rating Scale (HAM-A), and the Hamilton Depression Rating Scale (HAM-D). L-tryptophan (TRP) and its metabolites: 5-hydroxyindoleacetic acid (5-HIAA), kynurenine (KYN), xanthurenic acid (XA) and quinolinic acid (QA) were measured in urine by liquid-chromatography-tandem mass spectrometry and related to creatinine level. Patients with SIBO were recommended to take rifaximin for 10 days at daily dose 1200 mg, and this cycle was repeated in subsequent two months.

**Results:**

Mild and moderate anxiety, as well as mild depression were diagnosed in all SIBO patients. Changes in TRP metabolism were also observed in these patients. Specifically, an increase in the activity of the serotonin pathway of TRP metabolism in the group SIBO-D was observed. The SIBO-C patients showed an increase in the concentration of KYN, XA and QA. 5-HIAA/TRP and KYN/TRP ratios significantly decreased in group SIBO-D, and KYN and QA levels decreased in group SIBO-C after treatment with rifaximin. The levels of anxiety and depression decreased in both groups.

**Conclusion:**

Rifaximin treatment of SIBO patients ameliorated their mood disorders and gastrointestinal aliments underlined by changes in tryptophan metabolism.

*Trial registration* Retrospectively registered (if applicable).

## Introduction

Optimal composition of intestinal bacteria is an essential condition for good health, including mental state. The gut microbiota plays digestive, protective, metabolic and immuno-regulatory functions [1, 2]. The normal small bowel has lower levels of bacteria colonization compared with the colon. This balance is altered in patients with small intestinal bacterial overgrowth (SIBO).

The composition of bacterial flora in the intestine is changed by various factors, including antibiotics, non-steroidal anti-inflammatory drugs, improper diet and others [3]. Chronic stress also leads to changes in the small intestinal environment, which creates unfavorable conditions for commensal bacteria, and consequently increased translocation of bacteria from the large intestine. Even non-pathogenic species can cause changes in the digestive tract. [4, 5].

Clinical picture of SIBO is diverse, but often similar to irritable bowel syndrome (IBS). This coincidence leads to recognition of the bacterial theory of IBS pathogenesis [6]. It assumes that the primary cause of IBS is external infection or the growth of endogenous bacteria, which secondary leads to the clinical manifestation of IBS. Some beneficial results of antibiotic treatment also confirm this assumption. Rifaximin, as a eubiotic, has proven to be useful for this purpose [7].

The aim of the present study was to assess the mental state of patients with small intestinal bacterial overgrowth in relation to tryptophan metabolism and rifaximin treatment.

## Methods

### Patients

The study involved 120 subjects, aged 23–61 years, enrolled in 2017–2021, selected out of 1260 patients who were tested for the presence of small intestinal bacterial overgrowth (SIBO). Three groups were identified, 40 individuals each. Group Control included subjects without any complaints, and with a negative result of the hydrogen breath test (Control). Group SIBO-D consisted of patients with chronic diarrhea, and with a positive result of the hydrogen breath test, whereas group SIBO-C – patients with chronic constipation, and with a positive result of the hydrogen breath test. The SIBO-D group was characterized by loose or watery stools, occurring > 25% of the time, at least for six months. In the SIBO-C group there were two or fewer bowel movements a week and hard and lump stools for a minimum six months. Moreover, all patients in both SIBO groups suffered from abdominal pain, bloating, as well as anxiety and depressed mental mood.

All individuals were recommended to record the type and quantity of nutrients consumed for 14 days prior to the investigations in the nutritional diary. The average TRP intake was calculated using the nutritional calculator with application Kcalmar.pro-Premium (Hermex, Lublin, Poland). The hydrogen breath test was performed using a Gastrolyzer (Bedfont, Ltd, Harrietsham, UK)., a fasting level of hydrogen was measured in expired air, after which 10 g lactulose dissolved in 200 mL water was administrated and breath samples were collected immediately and at 15-min minute intervals for 3 h. The criterion for SIBO positive diagnosis was a minimum of 20 ppm of hydrogen within the first 90 min of testing.

At the outset, the patients assessed themselves their mental health, and then each of them was assessed for mental condition using The Hamilton Anxiety Rating Scale (HAM-A), and the Hamilton Depression Rating Scale (HAM-D). European standards have been adopted for both scales: 10–19 points – mild anxiety/depression, 19–29 points – moderate anxiety/depression, over 30 points – severe anxiety/depression. The Gastrointestinal Symptom Rating Scale was used to assess abdominal complaints. To determine other diseases of the GI tract all patients underwent endoscopic and histological examination of gastric, duodenal, small intestinal and colonic mucosa. The following exclusion criteria were applied: H-pylori-induced gastritis, lymphocytic and ulcerative colitis, Crohn disease, allergy and food intolerance, liver and renal diseases, diabetes, and the use antibiotics, probiotics and psychotropic drugs in the month prior to enrolment into the study.

### Laboratory tests

The following laboratory tests were performed: blood cell count, C-reactive protein, glucose, bilirubin, urea, creatinine, profile of lipids, thyroid-stimulating hormone, free thyroxine, free triiodothyronine, alanine and asparagine aminotransferases, gamma-glutamyltranspeptidase, alkaline phosphatase, amylase, lipase, antibodies to tissue transglutaminase, deaminated gliadin peptide, and fecal calprotectin.

Urine samples for the analysis of TRP and its metabolites were collected at the morning on an empty stomach into a special container with a solution of 0.1% hydrochloric acid as a stabilizer. Using liquid chromatography with tandem mass spectrometry (LC–MS/MS, Ganzimmun Diagnostics AG, Mainz, Germany), we determined the concentration of TRP and its metabolites: 5-hydroxyindoleacetic acid (5-HIAA), L-kynurenine (KYN), xanthurenic acid (XA), and quinolinic acid (QA). The levels of these metabolites were expressed in mg per gram of creatinine (mg/gCr). The ratios of the levels of 5-hydroxyindoleacetic acid and tryptophan as well as KYN and TRP were also calculated. The 5-HIAA/TRP ratio was considered as an exponent of the activity of the serotonin pathway and the KYN/TRP ratio reflected the activity of the kynurenine pathway in tryptophan metabolism.

### Therapeutic procedures

Treatment with antibiotic was recommended when clinical, laboratory, and breath testing were completed. Patients with SIBO (groups SIBO-D and SIBO-C) were recommended to take rifaximin, in daily dose 1200 mg for 10 days. This cycle was repeated three times at monthly intervals. It was also recommended to maintain a balanced diet containing similar nutritional ingredients without any other medications, probiotics or dietary supplements. Follow-up medical examinations with the assessment of the relief of somatic and psychic symptoms were performed after 1, 2 and 3 months. Laboratory and breath tests were repeated after the end of the third treatment cycle (the fourth month). The studies were conducted as an open-label therapeutic procedure.

### Data analysis

Normality of data distribution was checked using Shapiro–Wilk W test. U Manna-Whitney's test was used to compare difference between two groups. Dunn's multiple comparisons test following The Kruskal–Wallis test was used to compare three groups. Differences within groups before and after treatment were analyzed by Wilcoxon signed-rank test. All statistical analyses were performed with STATISTICA 13.3 software (TIBCO Software Inc., Palo Alto, CA, USA).

## Results

There were no significant differences in the distribution of age, gender, BMI, ALT and AST activity, as well as glomerular filtrating ratio between SIBO patients and controls (Table 1). The concentration of the C-reactive protein was higher in both SIBO-D and SIBO-C patients than controls (*p* < 0.01). The concentration of fecal calprotectin was higher in SIBO-D patients as compared to controls (*p* < 0.01). There were no significant differences in the fasting results of the lactulose hydrogen breath test between SIBO-D and SIBO-C: 68.01 ± 15.9 vs 57.4 ± 13.4 (*p* > 0.05). There were no differences in average daily intake of L-tryptophan between any pair of the groups (Table 1). Normal values of ALT, AST and GFR indicate that the individuals enrolled in the study did not suffer from any liver or kidney disease that could affect TRP metabolism, changing urinary excretion of its products.

**Table 1:** Characteristics of the subjects enrolled in the study and results of laboratory tests: Controls, patients with small intestinal bacterial overgrowth and diarrhea predominant (SIBO-D), patients with small intestinal bacterial overgrowth and constipation predominant (SIBO-C); Mean ± SD

Feature	Control	SIBO-D	SIBO-C	*p*
Age (years)	43.9 ± 5.3	47.4 ± 8.5	48.3 ± 12.6	Ns
Gender – M/F	19/21	17/23	15/25	Ns
BMI (kg/m^2^)	23.7 ± 1.8	22.9 ± 1.1	25.2 ± 2.4	Ns
GFR (ml/min)	101.3 ± 9.6	102.6 12.8	98.1 ± 10.6	Ns
ALT (U/L)	14.2 ± 2.6	19.4 ± 8.7	19.6 ± 12.5	Ns
AST (U/L)	12.9 ± 3.1	18.2 ± 5.8	21.7 ± 8.2	Ns
CRP (mg/L)	1.24 ± 0.32	7.16 ± 1.32	2.92 ± 1.46	< 0.01^a^
FC (µg/g)	21.8 ± 7.32	48.8 ± 18.3	32.6 ± 16.4	< 0.01^a^
LHBT (ppm)	10.3 ± 3.62	68.1 ± 15.9	57.4 ± 13.4	< 0.001^a^
TRP (mg/24 h)	1296 ± 198	1139 ± 162	1306 ± 301	Ns

M Male, F Female, *BMI* Body mass index, *GFR* Glomerular filtrating ratio, *ALT* Alanine aminotransferase, *AST* Aspartate aminotransferase, *CRP* C-reactive protein, *FC* Fecal calprotectin, *LHBT* Lactulose hydrogen breath test, *TRP* L-tryptophan, Ns – *p* > 0.05, differences between groups: a – group C vs group SIBO-D

**Fig. 1:**
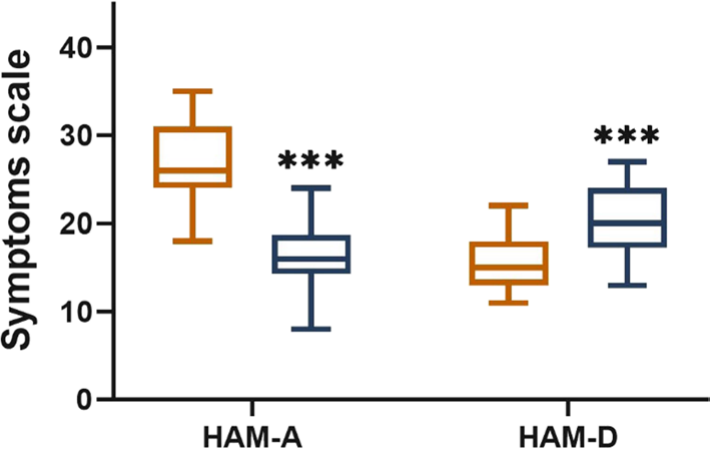
Hamilton Anxiety Rating Scale (HAM-A), and Hamilton Depression Rating Scale (HAM-D) in patients with SIBO with diarrhea predominant (SIBO-D, orange) and with constipation predominant (SIBO-C, grey); median with boxes represent I and III quartiles, and error bars represent the minimum and maximum values. Differences between groups were analyzed by U Manna-Whitney's test; *n* = 40 in both groups; *** *p* < 0.001

Based on the adopted criteria (HAM-A and HAM-D) there was mild anxiety in 12 patients with SIBO-D (30.0%), a moderate anxiety in 22 (55.5%) patients, and severe anxiety in 6 (15.0%) patients. In this group mild depression was diagnosed in 16 (40.0%) patients, and moderate depression in 24 (60.0%) patients. However, anxiety predominated in the SIBO-D group, while symptoms of depression – in SIBO-C patients. In SIBO-C mild anxiety was diagnosed in 26 (65.0%) patients, and moderate anxiety in 14 (35.0%) patients. In addition, mild depression was found in 21 (52.5%) patients, and moderate depression in 19 (47.5%) people. The mean scores for anxiety were 9.85 ± 2.18 in group C, 26.5 ± 4.61 in group SIBO-D, and 16.4 ± 6.12 in group SIBO-C; the difference between group SIBO-D and group SIBO-C was significant (*p* < 0.001). The mean depression test scores were 7.22 ± 1.90 in Controls, 15.5 ± 3.39 in group SIBO-D, and 20.3 ± 3.92 in group SIBO-C, the difference between groups SIBO-D and SIBO-C was statistically significant (*p* < 0.001, Fig. 1).

We found differences between group SIBO-D and SIBO-C in the urinary level of tryptophan and its metabolites (Table 2). The urinary levels of TRP in group SIBO-C were lower than in the control group. The levels of 5-HIAA in group SIBO-D were higher compared to the other groups. However, the 5-HIAA/TRP ratio was significantly higher in patients with group SIBO-D as compared to controls, and SIBO-C. KYN levels, and the KYN/TRP ratio were higher in both groups with SIBO, compared to the controls. The urinary level of XA and QA were highest in patients with SIBO-C and both SIBO groups had it significantly higher than in the control group.

**Table 2:** Urinary excretion of tryptophan and its metabolites in healthy subjects (group Control), and in patients with small intestinal bacterial overgrowth and with diarrhea-predominant (SIBO-D) or with constipation-predominant ( SIBO-C); average ± SD

Feature	Control	SIBO-D	SIBO-C	*p*
TRP (mg/gCr)	13.3 ± 2.30	12.2 ± 2.31	10.1 ± 1.22	< 0.001^b^
5-HIAA (mg/gCr)	3.01 ± 0.39	3.89 ± 0.34	2.56 ± 0.64	< 0.05^a,c^
5-HIAA/TRP	0.24 ± 0.01	0.32 ± 0.091	0.258 ± 0.016	< 0.01^a,c^
KYN (mg/gCr)	0.45 ± 0.11	0.58 ± 0.13	0.83 ± 0.18	< 0.001^b,c^
KYN/TRP	0.036 ± 0.011	0.04 ± 0.012	0.083 ± 0.023	< 0.001^b,c^
XA (mg/gCr)	0.68 ± 0.27	0.81 ± 0.24	0.98 ± 0.25	< 0.05^a,b,c^
QA (mg/gCr)	3.02 ± 0.99	4.17 ± 0.97	6.98 ± 0.94	< 0.05^a^

TRP Tryptophan, 5-HIAA—5-hydroxyindoleacetic acid, KYN Kynurenine, XA Xanthurenic acid, QA Quinolinic acid, differences between groups: ^a^Control vs SIBO-D, ^b^Control vs SIBO-C, ^c^SIBO-D vs SIBO-C

**Fig. 2:**
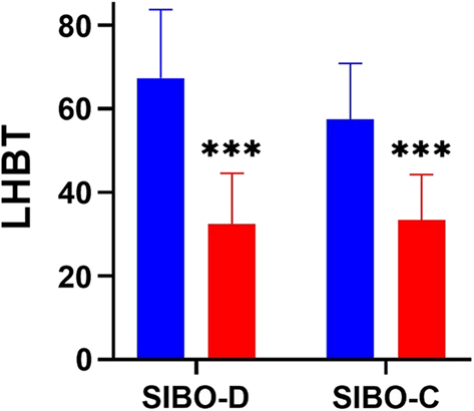
Lactulose hydrogen breath test (LHBT) in patients with low mood scores with chronic diarrhea (SIBO-D) and chronic constipation (SIBO-C) before (blue) and after rifaximin treatment (red); bars represent mean, error bars represent standard deviation. Differences in both groups before and after treatment were tested by Wilcoxon signed-rank test; *n* = 40 in both groups; *** *p* < 0.001

After rifaximin treatment average results of LHBT test decreased significantly in both groups, from 67.3 ± 16.3 to 32.3 ± 12.2 ppm in group SIBO-D, and from 57.4 ± 13.4 to 32.3 ± 10.9 ppm in group SIBO-C (Fig. 2).

**Fig. 3:**
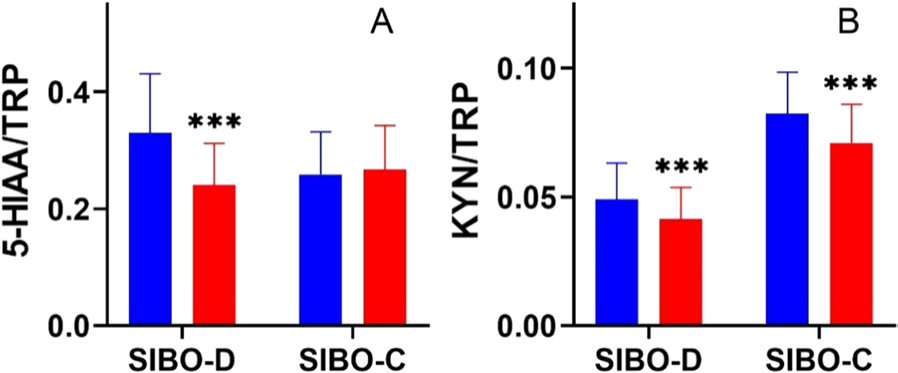
Ratio between urinary levels of **A** tryptophan and 5-hydroxyaminoacetic acid (TRP/5-HIAA) and **B** tryptophan and L-kynurenine (TRP/KYN) in patients with low mental mood with chronic diarrhea (SIBO-D) and chronic constipation (SIBO-C) before (blue) and after rifaximin treatment (red); boxes represent mean, error bars represent standard deviation. Differences in both groups before and after treatment were evaluated by Wilcoxon signed-rank test; *n* = 40 in both groups; *** *p* < 0.001. Schemes follow the same formatting

At the same time, the ratio of 5-HIAA/TRP decreased from 0.329 ± 0.101 to 0.240 ± 0.071 in group SIBO-D whereas it remained similar in group SIBO-C. The ratio of KYN/TRP also decreased from 0.049 ± 0.014 to 0.041 ± 0.012 in group SIBO-D and from 0.082 ± 0.016 to 0.071 ± 0.015 in group SIBO-C (Fig. 3).

**Fig. 4:**
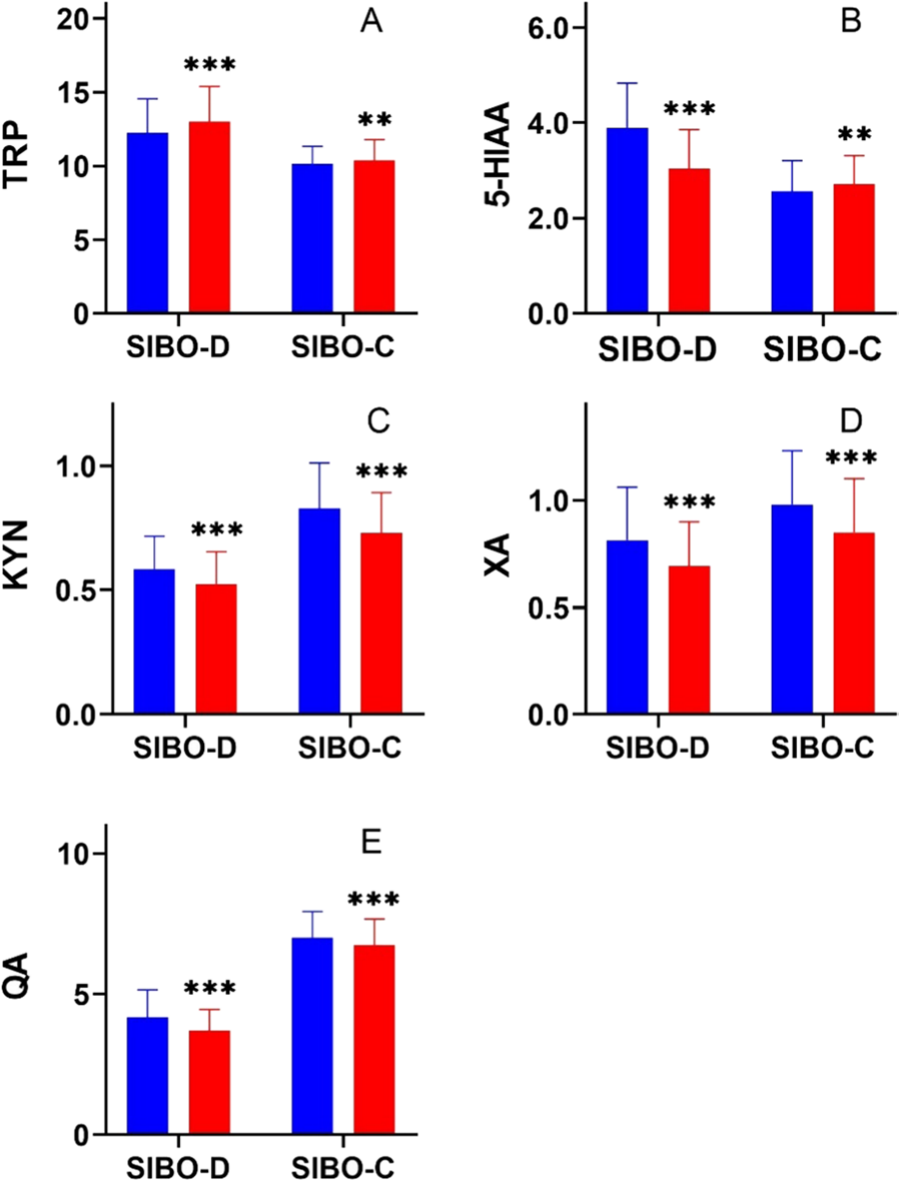
Urinary levels of **A** tryptophan (TRP), **B** 5-hydroxyaminoacetic acid (5-HIAA), **C** L-kynurenine (KYN), **D** xanthurenic acid (XA) and **E** quinolinic acid (QA) expressed in milligram per gram of creatinine (mg/gCr) in patients with low mental mood with chronic diarrhea (SIBO-D) and chronic constipation (SIBO-C) before (blue) and after rifaximin treatment (red); bars represent mean, error bars represent standard deviation. Differences in both groups before and after treatment were examined by Wilcoxon signed-rank test; *n* = 40 in both groups; *** *p* < 0.001

The urinary levels of TRP and its metabolites also decreased after rifaximin treatment in both groups (Fig. 4). In group SIBO-D TRP levels increased from 12.2 ± 2.31 mg/gCr to 13.3 ± 2.39 mg/gCr while its metabolites significantly decreased after treatment: 5-HIAA—3.89 ± 0.94 vs 3.04 ± 0.82 mg/gCr, KYN—0.58 ± 0.13 vs 0.52 ± 0.12 mg/gCr, XA – 0.81 ± 0.25 vs 0.69 ± 0.69 ± 0.21 mg/gCr, and QA—4.17 ± 0.98 vs 3.69 ± 0.75 mg/gCr. Similar significant changes were found in group SIBO-C: KYN – 0.58 ± 0.13 vs 0.52 ± 0.12 mg/gCr, XA – 0.98 ± 0.25 vs 0.84 ± 0.24 mg/gCr, QA – 6.98 ± 0.65 vs 6.72 ± 0.33 mg/gCr.

**Fig. 5:**
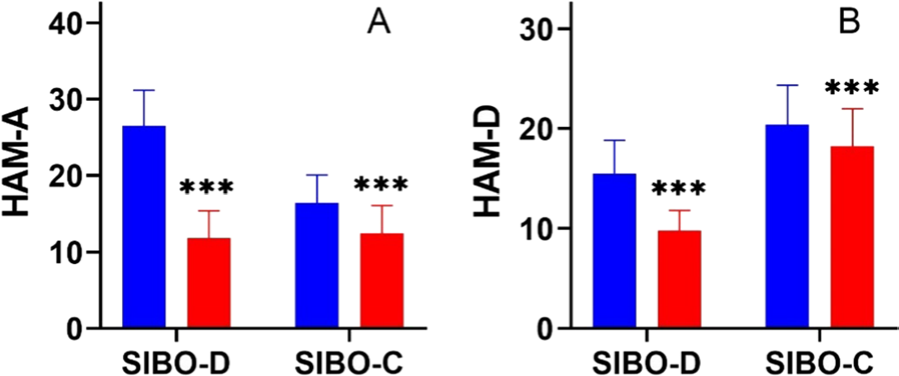
The Hamilton Anxiety Rating Scale (HAM-A) **A**, and the Hamilton Depression Rating Scale (HAM-D) **B** in patients with small intestinal bacterial overgrowth with diarrhea predominant (SIBO-D) and with constipation predominant (SIBO-C) before (blue) and after rifaximin treatment (red); bars represent mean, error bars represent standard deviation. Differences in both groups before and after treatment were examined by Wilcoxon signed-rank test; *n* = 40 in both groups; *** *p* < 0.001

Anxiety and depression levels were also ameliorated by rifaximin treatment in both SIBO groups (Fig. 5). In group SIBO-D – anxiety symptoms score decreased from 26.5 ± 4.61 to 11.8 ± 3.56 and depression scores from 15.5 ± 3.33 to 9.82 ± 2.01 (*p* < 0.001). In group SIBO-D anxiety score decreased from16.4 ± 3.62 to 12.5 ± 3.59 and depression score from 20.3 ± 3.97 to 18.2 ± 3.74 (*p* < 0.001). Nevertheless, the level of mental disorders in the SIBO group was still slightly higher than in the control group.

Rifaximin was well tolerated, and three months after its administration no patient had diarrhea, and only 3 (7.5%) patients with SIBO-C still had mild abdominal pain, constipation and bloating.

## Discussion

Small intestinal bacterial overgrowth is common in all populations and its clinical manifestations are diverse. Most often the lactulose hydrogen breath test is used to confirm the diagnosis. The concentration of hydrogen in expired air more than 20 ppm in the first 90 min during the test is the criterion for SIBO occurrence., In about 15% of people SIBO is asymptomatic, but it may cause abdominal pain and abnormal stool bowel movements. However, it is not known why diarrhea dominates in some patients and constipation in others. Presumably the different species of bacteria and/or their proportion can have a significant impact on dissimilarity of symptoms.

Many studies have shown the influence of the microbiome on central nervous system and a communication pathway between gut microbiome and the brain [8, 9]. For this reason, the term “the gut-brain axis” has been replaced with “the microbiome-gut-brain axis” [10]. Functional mechanisms behind the axis are not fully understood. It is assumed that some biologically compounds, including serotonin, send signals trough the vagus nerve to the brain, with information about changes in the gut microbiome. In turn, signals sent from the brain affect the state of the microbiome [11]. Some strains of the microbiome may affect the nervous system by increasing production of neurotransmitters [12]. The properties of these bacteria are used as psychobiotics in clinical practice [13]. This justifies their use in patients with IBS, who often suffer from anxiety and depression, but no consistent results in the treatment of this comorbidity were obtained [14–16].

It is also still unclear whether the abdominal complaints in patients with IBS are a cause of mental disorders or their consequence. This justifies the studies on the role of dietary compounds in the pathogenesis of psychosomatic diseases. Tryptophan and its metabolites play a special role. Serotonin is synthesized in the gastrointestinal (GI) tract and in the central and visceral neurons system and its homeostasis is important for a proper functioning of the gut-brain axis [17]. The increase in serotonin synthesis in gut is a consequence of overexpression of tryptophan hydroxylase (TPH-1) or inhibition of the activity of inhibitors of its degradation. Serotonin synthesis can be affected by food and bacterial factors [18]. In turn, bacterial toxins can inhibit the tissue catabolism of serotonin, but may also reduce its synthesis by directing exogenous TRP into kynurenine pathway, with participation of indole 2,3-dioxygenase (IDO-1) [19]. Similar process occur in the brain with the involvement of isoenzymes TPH-2 and IDO-2 [20, 21].

Some kynurenine pathway metabolites, such as KYN and QA show neurotoxic affects with clinical implications [22]. The imbalance between serotonin and KYN metabolic pathways of TRP was considered as a factor of depression pathogenesis [23]. The role of bacteria in these complex processes is still poorly known, which inspired us to undertake our research.

Brain neurotransmission may be disrupted by various neurotoxic factors, including TRP metabolites [24]. These events are mainly attributed to KYN and QA [25]. The CNS receives about 60% of L-kynurenine from the periphery trough the transport across the blood–brain barrier, while the rest is produced locally. The metabolism of TRP trough the kynurenine pathway occurs mainly in the blood, lymphoid tissue and also in the digestive tract [26]. In previous studies with SIBO patients, we found an increase in the number of intraepithelial lymphocytes (IELs) in the small intestine [27], which can be a source of kynurenine compounds. All subpopulations of active IELs can migrate from the intestinal wall to another organ, including centrally nervous system. As a consequence, the blood–brain barrier may be weakened and the transport of kynurenine compounds to the CNS may be facilitated. Such consequences are assumed by the inflammatory concept of depression. Nevertheless, the mechanisms of this adversely effect of kynurenine compounds on the nervous system are not exactly known. Generally accepted opinion assumes that their toxic effects are related to production of free radicals and the negative influence of energy metabolism in nerve cells [28]. As a consequence of these changes, they cause disturbances in neurotransmission, as well as degeneration and apoptosis of neurons.

In our study, the profile of changes in tryptophan metabolism was slightly different in both SIBO groups. In SIBO-D patients the activity of the serotonin pathway was greater than in SIBO-C group. On the other hand, in the SIBO-C group higher activity of the kynurenic pathway was found. These differences may be the reason of the variation in abdominal symptoms, as well as the nature and severity of the mood changes.

Obtained results confirm an important role of TRP metabolism in the pathogenesis of gastrointestinal disorders. In other studies, Berstad et al. recognized TRP as an amino acid,,essential” for IBS pathogenesis [29]. Clarke et al. observed increases in the L-KYN levels and L-KYN/L-TRP ratio in male IBS patients as compared with healthy subjects [30]. The authors interpreted their results as the consequence of an increase of IDO, an enzyme responsible for TRP degradation in the kynurenine pathway. Kaszthelyi et al. showed that IBS patients had higher blood concentration of both serotonin and KYNA compared to healthy controls [31]. Christmas et al. suggested that diarrhea-predominant IBS patients would have elevated TRP in plasma due to alternations in its metabolism, mainly enhanced serotonin pathway and inhibited kynurenine pathway. They therefore hypothesized on possible enhanced serotonin activity in pathogenesis of IBS-D [32]. Fitzgerald et al. showed a positive correlation between the level of KYN/TRP and intensity of IBS symptoms, as well as with depressive symptoms [33].

Many studies confirmed the effectiveness of antibiotics in removing abdominal symptoms, but mental state assessment has received less attention. The obtained results indicate that during 3-months period of rifaximin treatment TRP metabolism has turned in the right direction.

Symptoms of anxiety and depression did not completely resolve after rifaximin treatment. This is due to the complex pathogenesis of mental disorders, as well as abdominal complaints. It cannot be ruled out that in some patients the mental disorders were endogenous, and that bacterial and food factors adversely affected the course of depression [34]. It is possible that increased expression of genes encoding pro-inflammatory cytokines may determine the genetic predisposition to mood disorders trough increased activation of the kynurenic pathway [35]. It can be assumed that the kynurenic pathway represents one of the main points of the interaction between genetic and environmental factors involved in the pathogenesis of depression.

Our study has two limitations. Firstly, the TRP intake in the last months prior to the start of the study was not established. Secondly, TRP consumption was not standardized during clinical trial. Therefore, further research is needed in this area with detailed diet control in SIBO patients.

## Conclusions

Bacteria that are involved in SIBO, cause an impairment in tryptophan metabolism, which, in turn, may induce gastrointestinal aliments, including diarrhea and constipation. Impaired TRP metabolism may also underline mood disorders, including mild and moderate anxiety and mild depression. Treatment with rifaximin reduced the bacterial population in the gastrointestinal tract, improved tryptophan metabolism reducing the amount of neurotoxic metabolites and decreased gastrointestinal aliments and mood disorders. Therefore, rifaximin may be considered in the prevention and treatment of SIBO-related complications.

## Data Availability

All data supporting reported results can be found at Department of Clinical Nutrition and Gastroenterological Diagnostics, Medical University of Lodz (jan.chojnacki@umed.lodz.pl).

## Abbreviations

SIBO-D: Small intestinal bacterial overgrowth with diarrhea
SIBO -C: Small intestinal bacterial overgrowth with constipation
HAM-A: Hamilton anxiety rating scale
HAM-D: Hamilton depression rating scale
LHBT: Lactulose hydrogen breath test
TRP: L-tryptophan
5-HIAA: 5-Hydroxyindoleacetic acid
KYN: Kynurenine
XA: Xanthurenic acid
QA: Quinolinic acid
